# Muscle Quality Index is inversely associated with psychosocial variables among Chilean adolescents

**DOI:** 10.1186/s12889-023-16978-w

**Published:** 2023-10-26

**Authors:** Guillermo Barahona-Fuentes, Álvaro Huerta Ojeda, Gabriela Lizana Romero, Pedro Delgado-Floody, Daniel Jerez-Mayorga, María-Mercedes Yeomans-Cabrera, Luis Javier Chirosa-Ríos

**Affiliations:** 1 Department of Physical Education and Sport, Faculty of Sport Sciences, University of Granada, Granada, Spain; 2 Núcleo de Investigación en Salud Actividad Física y Deporte ISAFYD, Universidad de Las Américas, Sede Viña del Mar, Chile; 3 Faculty of Education and Social Sciences, Universidad Andres Bello, Viña del Mar, Chile; 4 Department of Physical Education, Sport and Recreation, Universidad de La Frontera, Temuco, 4811230 Chile; 5 Exercise and Rehabilitation Sciences Institute, School of Physical Therapy, Faculty of Rehabilitation Sciences, Universidad Andres Bello, 7591538 Santiago, Chile; 6 Facultad de Salud y Ciencias Sociales, Universidad de Las Américas, Viña del Mar, Chile

**Keywords:** Muscle quality, Depression, Anxiety, Stress, Adolescence

## Abstract

A good muscle quality index (MQI) may have an inverse relationship with psychosocial variables of depression, anxiety, and stress in adolescents. Unfortunately, little scientific evidence has related MQI to psychosocial variables in this population. Therefore, this research aimed to determine the relationship between the MQI and psychosocial variables of depression, anxiety, and stress in Chilean adolescents. In this quantitative correlational design study, sixty adolescents participated voluntarily (mean ± standard deviation [SD]: age 15.11 ± 1.78 years). Anthropometric parameters, prehensile strength, MQI, and psychosocial variables were evaluated. The results showed that adolescents with high levels of MQI presented lower levels of depression (7.50 ± 6.06 vs. 10.97 ± 5.94), anxiety (5.64 ± 4.81 vs. 9.66 ± 5.12), and stress (6.79 ± 5.09 vs. 10 ± 5.58), in addition to reported lower abdominal obesity (WtHR, 0.47 ± 0.07 vs. 0.52 ± 0.07) than those with low levels of MQI. The group with high levels of MQI reported a higher prevalence of nonanxiety (81.3%, *p* = 0.031) and a lower prevalence of abdominal obesity (55.8%, *p* = 0.023). Likewise, a significant inverse association was evidenced between MQI and depression (β; -6.18, 95% CI; -10.11: -2.25, *p* = 0.003), anxiety (β; -6.61, 95% CI; -9.83: -3.39, *p* < 0.001) and stress (β; -4.90, 95% CI; -8.49: -1.32 *p* = 0.008). In conclusion, the results suggest that high levels of MQI are associated with a higher prevalence of nonanxiety in adolescents and a significant inverse association between MQI and levels of depression, anxiety, and stress.

**Supplementary Information:**

The online version contains supplementary material available at 10.1186/s12889-023-16978-w.

## Introduction

Currently, alterations in psychosocial variables such as depression, anxiety, and stress affect different segments of the population [1–3], causing a decrease in quality of life [3]. Likewise, it has been shown that people with psychosocial disorders have a 10–15 year decrease in life expectancy compared to the general population [4]. In this regard, it has been shown that high levels of anxiety, stress, and depression have occurred in adults, children, and adolescents [5]. It has been observed that most adults with psychosocial disorders begin their symptoms in childhood or adolescence [6]. Indeed, a recent meta-analysis showed that the first psychosocial disorders appear in adolescence, shortly before the age of 14 [7]. Therefore, it is essential to detect and monitor any type of psychosocial disorder early in any population segment.

For the management and control of psychosocial variables, nonpharmacological methods have been used [8], medication [9], physical activity, and physical exercise [10]. The latter has become a determining factor in reducing depression, anxiety, and stress [11, 12]. An example is the study developed by Delgado-Floody et al. [13]. They associated depression with low levels of physical activity, high obesity, and dissatisfaction with body image in Chilean preadolescents. They concluded that schools should promote physical activity to improve psychological and physical health in preadolescents, which would reduce future mental illnesses. In this sense, scientific evidence shows that physical activity habits are strongly associated with mental well-being [14]. Indeed, a decrease in psychosocial variables experienced through the practice of physical activity and exercise would be mediated by the different types of adaptations that the organism would undergo [15], for example, brain development [15], which in turn would condition psychosocial variables [16] by decreasing depression, anxiety, and stress [15, 17]. When examining physical activity and exercise interventions, it has been documented that most treatments are based on aerobic exercise [17, 18]. Likewise, treatments and research relating strength development to psychosocial variables have been scarce [12, 19]. In this sense, a recent meta-analysis conducted by Barahona-Fuentes et al. [12] showed that good strength development, regardless of the methodology used, would allow control of anxiety and depression levels in adolescents. However, these same authors concluded that this field was not investigated in depth [12].

Indeed, a direct relationship between muscle strength and the muscle quality index (MQI) has been demonstrated, and it has been determined that a low level of strength will trigger a poor MQI [20–22]. Likewise, it has been shown that MQI is also influenced by high subcutaneous adipose tissue content and high-fat percentages, with an inverse relationship between MQI and fat tissue [23, 24]. In contrast, high levels of MQI play a fundamental role in preventing chronic diseases [25]. In this sense, Lee et al. [26] examined the relationship between insulin sensitivity and muscle quality in adolescents and found that muscle quality is strongly associated with insulin sensitivity.

In light of the challenges posed by psychosocial variables, exploring possible mitigating factors is imperative. One promising avenue of research is the role of physical health, particularly the muscle quality index (MQI), as a potential countermeasure to the adverse effects of depression, anxiety, and stress in adolescents. However, despite the critical importance of this topic, there remains a lack of scientific research directly relating MQI in adolescents to psychosocial variables [12].

Therefore, this study aims to determine the intricate relationship between the MQI and psychosocial variables of depression, anxiety, and stress in the Chilean adolescent population. Our hypothesis postulates a significant inverse association between the MQI and the prevalence of depression, anxiety, and stress in adolescents. By exploring this relationship, we aim to expand the evidence on a critical aspect of adolescent mental health and contribute to the existing scientific knowledge on this topic.

## Materials and methods

The following study was conducted in accordance with the Strengthening the Reporting of Observational Studies in Epidemiology (STROBE) guidelines and recommendations [27, 28].

### Research design and procedure

**Fig. 1:**
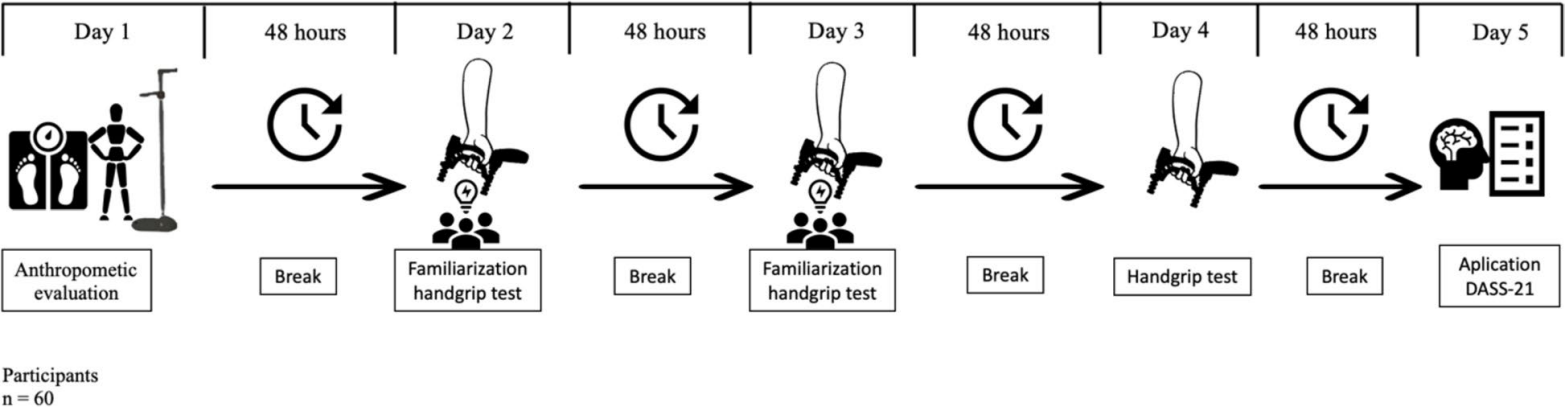
Study protocols

The study had a quantitative correlational design. To start the investigation, on April 4, 2022, an invitation was extended to two schools in the V region of Chile. However, because of the COVID-19 pandemic, only one agreed to take part in the investigation. On July 27, 2022, the school that wanted to participate had a total of 74 adolescent students. Of these, 14 did not want to be part of the study. Therefore, the sample consisted of 60 adolescents who voluntarily agreed to be part of the study. Convenience sampling was employed to recruit participants due to the practicality and accessibility of this method. Each participant attended the measurement center for five days at 48-h intervals. During the first visit, basic anthropometric evaluations were performed. The second and third days were intended to familiarize the participants with the prehensile strength test. On the fourth day, the prehensile strength test was evaluated. On the fifth day, psychosocial variables were assessed through the Depression Anxiety Stress Scales (DASS-21) (Fig. 1). Likewise, they were asked not to perform physical activity during evaluation days to avoid a decrease in physical performance. All the tests proposed in the study were performed bilaterally, starting with the participants' dominant limb.

### Participants

We used statistical software (G*Power, v3.1.9.7, Heinrich-Heine-Universität, Düsseldorf, Germany) to determine the appropriate sample size [29]. The combination of tests used in the statistical software to calculate the sample size was as follows: (a) x^2^ tests, (b) goodness-of-fit tests: Contingency tables y (c) a priori: Compute required sample size – given α, power, and effect size. Tests considered two tails, effect size w = 0.47, α-err prob = 0.05, power (1-β err prob) = 0.8, and Df = 5. The total sample size was 59 participants.

The sample consisted of 60 adolescents (26 females and 34 males) (mean ± standard deviation [SD]: age 15.11 ± 1.78 years). All participants had to meet the following inclusion criteria: being adolescents between the ages of 13 and 17 who were enrolled in the establishment that accepted the invitation to the study, subjects must not have performed regular strength training (≥ 3 days per week) during the last year, being free of skeletal muscle injuries in the upper extremities during the tests, reading and signing an informed assent form, and having their guardians read and sign an informed consent form. Those who did not meet these criteria were excluded from the study.

### Ethics approval and consent to participate

The informed consent was obtained from all the participants and from the legal guardians of the participants who were below 17 years of age. The study was approved by the Scientific Ethics Committee of the Universidad de Las Américas (project ID CEC_FP_2021028). All methods were carried out in accordance with relevant guidelines and regulations [30].

### Anthropometry

Height (cm) was evaluated through a stadiometer from the feet to the vertex (Frankford plane). Weight (kg) and fat percentage (%) were assessed using a Tanita Inner Scan BC-554® digital scale. For weight, height, and fat percentage measurements, the adolescents were asked to wear shorts and a light T-shirt barefoot. BMI was determined by dividing kilograms of weight by the square of height in meters (kg/m2). Waist circumference (WC) was measured using a Seca® tape measure model 201 (Hamburg, Germany) at the height of the umbilical scar [31]. The waist-to-height ratio (WtHR) was obtained by dividing the WC by height. It was used to estimate the fat accumulation in the central zone of “abdominal obesity” of the body, following international standards [32]. In line with recent evidence, a cutoff of ≥ 0.54 was optimal to consider the cardiometabolic risk for the Latin American region [33].

### Prehensile strength test

Before starting the test, for 5 min, each participant performed a standardized warm-up—upper limb dynamic movements. Subsequently, the participants stood with the shoulder adducted in neutral rotation. The elbow should be in 180° extension, and the forearm and wrist should be in a neutral position [34]. Then, the digital hand dynamometer (JAMAR Plus® + , USA) was placed in the participant's hand. Then, the investigator indicated the word "squeeze" to start the test and "relax" to finish. The maximum voluntary contraction of the prehensile strength test was 3–5 s. Each participant performed the test twice with each hand (first the right hand and then the left hand). There was a 120-s pause between each repetition and a 1-min rest before assessing the other limb. All participants received verbal support during the execution of the test. The average result of the two repetitions for each hand resulted in handgrip muscle strength (HGS). This was used for the characterization of sample 1.

### Muscle Quality Index

The MQI was calculated in the field by dividing the HGS by the body mass index (BMI) [33, 34]. The field test has been strongly correlated with a laboratory MQI [35]. Poor MQI was categorized as ≤ 50° and good MQI as > 50°.

### DASS-21 Questionnaire

The abbreviated version of the Depression Anxiety Stress Scales (DASS-21), which has been culturally and idiomatically adapted and exhibits reliability and validity in Chilean adolescents, was used [36–38]. Specifically, the Depression scale assesses dysphoria, meaninglessness, self-depreciation, lack of interest, and anhedonia. The Anxiety scale considers subjective and somatic symptoms of fear, autonomic activation, situational anxiety, and subjective experience of anxious affect. The Stress scale assesses persistent nonspecific arousal, difficulty relaxing, irritability, and impatience. All DASS-21 items are answered on a Likert scale (0 to 3 points) according to the presence and intensity of the symptoms in the last week. Each scale has seven items; its total score is calculated by summing all the items' punctuation, which may vary between 0 and 21 points [36].

### Availability of data and materials

The datasets generated and/or analysed during the current study are available in a supplementary file.

### Statistical analysis

Normal distribution was tested using the Kolmogorov‒Smirnov test. Values are presented as the mean and standard deviation (SD) for continuous variables. Differences between mean values according to the MQI group were determined using ANOVA and the chi-square test. A simple linear regression estimated the association between MQI and psychological variables with a 95% confidence interval (95% CI). Sex and age were included as covariables. All statistical analyses were performed with SPSS statistical software version 23.0 (SPSSTM Inc., Chicago, IL). The alpha level was set at *p* < 0.05 for statistical significance.

## Results

Table 1 shows the comparison according to the MQI. The high MQI group presented lower depression (7,50 ± 6,06 vs. 10,97 ± 5,94), anxiety (5,64 ± 4,81 vs. 9,66 ± 5,12) and stress (6,79 ± 5,09 vs. 10 ± 5,58) scores than the low MQI group. In addition, the high MQI had lower abdominal obesity (WtHR, 0,47 ± 0,07 vs. 0,52 ± 0,07).

**Table 1:** Comparison of variables according to muscle quality index

	High-MQI (*n* = 28)	Low-MQI (*n* = 32)	Total (*n* = 60)		
	Mean ± SD	Mean ± SD	Mean ± SD	F	*p*-value
MQI	15.36 ± 1.77	14.91 ± 1.80	15.12 ± 1.79	0.951	0.333
Body mass (kg)	68.29 ± 19.06	72.80 ± 17.30	70.70 ± 18.13	0.920	0.341
Height (cm)	169.11 ± 7.45	160.09 ± 6.37	164.30 ± 8.20	25.537	0.000
BMI (kg/m2)	23.64 ± 5.33	28.20 ± 5.42	26.07 ± 5.80	10.734	0.002
Body fat (%)	22.00 ± 9.65	34.52 ± 7.55	28.67 ± 10.59	31.714	0.000
Depression	7.50 ± 6.06	10.97 ± 5.94	9.35 ± 6.20	4.991	0.029
Anxiety	5.64 ± 4.81	9.66 ± 5.12	7.78 ± 5.33	9.704	0.003
Stress	6.79 ± 5.09	10.00 ± 5.58	8.50 ± 5.56	5.369	0.024
WtHR (WC/size)	0.47 ± 0.07	0.52 ± 0.07	0.50 ± 0.07	11.491	0.001

*BMI* Body mass index, *kg* Kilograms, *MQI* Muscle quality index, *SD* Standard deviation, *WC* Waist circumference, *WtHR* Waist-to-height ratio

Data are shown as the mean and SD

The High-MQI group reported a higher prevalence of no anxiety (81.3%, *p* = 0.031) and a lower prevalence of abdominal obesity (55.8%, *p* = 0.023) (Table 2).

**Table 2:** The proportion of variables according to MQI

		CatMQI		Total	*p*-value
		High-MQI	Low-MQI		
Depression	No	11	4	15	*p* = 0.175
		73.3%	26.7%	100.0%	
	Low	3	5	8	
		37.5%	62.5%	100.0%	
	Moderate	5	9	14	
		35.7%	64.3%	100.0%	
	Severe	4	4	8	
		50.0%	50.0%	100.0%	
	Extreme	5	10	15	
		33.3%	66.7%	100.0%	
Anxiety	No	13	3	16	*p* = 0.031
		81.3%	18.8%	100.0%	
	Low	1	2	3	
		33.3%	66.7%	100.0%	
	Moderate	5	9	14	
		35.7%	64.3%	100.0%	
	Severe	2	3	5	
		40.0%	60.0%	100.0%	
	Extreme	7	15	22	
		31.8%	68.2%	100.0%	
Stress	No	18	11	29	*p* = 0.176
		62.1%	37.9%	100.0%	
	Leve	4	7	11	
		36.4%	63.6%	100.0%	
	Moderate	2	2	4	
		50.0%	50.0%	100.0%	
	Severe	2	7	9	
		22.2%	77.8%	100.0%	
	Extreme	2	5	7	
		28.6%	71.4%	100.0%	
Abdominal Obesity	No	24	19	43	*p* = 0.023
		55.8%	44.2%	100.0%	
	Abdominal Obesity	4	13	17	
		23.5%	76.5%	100.0%	

Data shown represent *n* (%)

Simple linear regression is shown in Table 3. An inverse association was observed between MQI and depression (β; -6.18, IC 95%; -10.11: -2.25, *p* = 0.003), anxiety (β; -6.61, IC 95%; -9.83: -3.39, *p* < 0.001) and stress (β; -4.90, IC 95%; -8.49: -1.32 *p* = 0.008) (Table 3). When variables were adjusted for sex and age, the significant values for anxiety were maintained (*P* = 0.006).

**Table 3:** Association between muscle quality index with psychosocial variables

				Standardized Coefficients	SE	95% Confidence Interval for B		*p*-value
	B	95%CI	Upper Bound	Beta		Lower Bound	Upper Bound	
Depression	-6.18	-10.11	-2.25	-0.38	1.96	-10.11	-2.25	*P* = 0.003
*	-4.14	-9.28	0.99	-0.26	2.56	-9.28	0.99	*P* = 0.112
Anxiety	-6.61	-9.83	-3.39	-0.47	1.61	-9.83	-3.39	*p* < 0.001
*	-6.02	-10.28	-1.77	-0.43	2.12	-10.28	-1.77	*P* = 0.006
Stress	-4.90	-8.49	-1.32	-0.34	1.79	-8.49	-1.32	*P* = 0.008
*	-2.66	-7.31	1.99	-0.18	2.32	-7.31	1.99	*P* = 0.257

Data shown represent β and 95%CI. *Represent variable adjusted by sex and age

## Discussion

The study's main objective was to determine the relationship between the MQI and psychosocial variables of depression, anxiety, and stress in Chilean adolescents. The main findings of this study were as follows: adolescents with high MQI levels evidenced (i) a lower score of depression, anxiety, and stress, in addition to lower abdominal obesity, compared to adolescents who presented lower MQI levels; (ii) the group with high MQI levels reported a higher prevalence of nonanxiety and a lower abdominal obesity prevalence; and (iii) a significant inverse association was evidenced between MQI with depression, anxiety, and stress.

### Psychosocial variables in Chilean adolescents

First, it was found that adolescents presented alterations in the psychosocial variables of depression (61.7% "moderate to extreme"), anxiety (68.3% "moderate to extreme"), and stress (33.3% "moderate to extreme"). In this sense, an alteration in these psychosocial variables has been associated with decreased academic performance, increased drug use and consumption, a higher prevalence of suicidal ideation [39], and a decrease in sports practice [13, 14]. In this line, recent meta-analyses have demonstrated the benefits of physical exercise on the levels of anxiety, stress, and depression [12, 17, 18], as well as the inverse association between high levels of physical fitness and low levels of psychosocial disorders [40–44]. In this context, scientific evidence has focused mainly on aerobic training and cardiorespiratory capacity as a modulator of psychosocial variables [18, 41, 43]. In parallel, the effects of strength training, its association with MQI, and its consequent impact on psychosocial variables are scarce [12]. Therefore, the antecedents presented in this research are pioneering in the association between MQI and psychosocial disorders in adolescents.

### Muscle strength development and its relationship with psychosocial variables

The results of the present study reported an inverse relationship between high MQI scores and depression (*p* = 0.003), anxiety (*p* < 0.001), and stress (*p* = 0.008). These results concord with recent meta-analyses that have determined associations between muscle strength development and psychosocial variables [12, 19, 45]. For example, Marques et al. [45] reported that muscle strength is inversely and significantly related to depression 0.85 in adults (95% CI: 0.80, 0.89). In parallel, in a meta-analysis developed by Barahona-Fuentes et al. [12], the effects of different modes of strength intervention on depression, anxiety, and stress in adolescents were found, evidencing a large and significant impact on depression (SMD = -1.61; CI = 95%: -2.54, -0.67, *p* = 0.0007) and anxiety (SMD = -1.75; CI = 95%: -0.03, -0.48; *p* = 0.007) [12]. Likewise, Whitworth et al. [46] described the beneficial effects of strength training in reducing posttraumatic stress levels in young adults. Similarly, several studies have been conducted in a population of older people, which have conclusively revealed that the level of basal prehensile strength is a protective factor against the development of depressive symptoms over time [47, 48]. However, it is relevant to note that these studies have focused on an older population and have exclusively addressed the basal level of manual grip strength. Despite this evidence, there is a notable absence of exploration of MQI, an essential component of muscle quality, which our research set out to examine in the context of Chilean adolescents. Based on the described antecedents, it has been evidenced that an increase in muscular strength decreases psychosocial variables [12, 19, 45]. In this context, it is understood that a higher level of strength would generate a higher MQI [20–22, 43]. Therefore, there should be a direct and inverse relationship between MQI and psychosocial variables.

### MQI and its relationship with psychosocial variables

In the present investigation, there was an inverse relationship between MQI and abdominal obesity in adolescents (*p* < 0.001). These results are in agreement with a recent study by Camaño-Navarrete et al. [49], who showed that subjects with a low MQI had greater abdominal obesity (*p* = 0.011) than the group with a high MQI. Likewise, these authors confirmed that the MQI is a partial mediator of the association between abdominal obesity and other variables, such as systolic blood pressure. In this context, it has been observed that MQI plays an essential role in preventing chronic diseases such as diabetes mellitus, cardiovascular risk, cancer, and abdominal obesity [25, 50, 51]. It has also been shown that adult men and women with high adipose tissue content have decreased muscle quality [23, 24]. Recently, Ikeue et al. [52] associated MQI with the accumulation of cardiovascular disease risk factors in obese patients and evidenced an accumulation of cardiovascular disease risk factors when combining WC and MQI, independent of sex and age [52]. Although our study did not evaluate cardiovascular risk factors since it was not the purpose of the study, it did assess abdominal obesity, which is directly associated with cardiometabolic risk markers. On the other hand, scientific evidence has also described that sedentary behavior can negatively influence the psychosocial variables of depression, anxiety, and stress [53]. An increase in sports practice through strength training—which would increase the MQI—could be related to a neural mechanism impacting brain development, specifically through an increase in hippocampal volume [16, 54]. This series of brain events associated with sports practice and increased MQI would cause a decrease in the psychosocial variables of depression, anxiety, and stress [16]. However, more research is needed to clarify this relationship [12].

### Limitations

Although the previously established sample size was met, we believe a larger sample size would allow us to extrapolate the data to the rest of the adolescent population. In this context, the COVID-19 pandemic restricted access to schools for external people. In addition, curricular progress in the teaching–learning process slowed down, causing less interest in scientific research during class hours from directors and parents. Despite this, the sample size studied allowed us to establish the relationship between MQI and psychosocial variables in adolescents. An emerging line of research has arisen in a population that had been neglected but is gradually becoming a priority in public policies in Chile and around the world.

## Conclusions

The MQI presents an inverse relationship with psychosocial variables of depression, anxiety, and stress, as well as with markers of cardiometabolic risk such as abdominal obesity. To the best of our knowledge, this was the first study to establish the relationship between MQI and psychosocial variables of depression, anxiety, and stress in the adolescent population. Future studies must verify these results through a larger sample size and test other important components through a strength training exercise intervention.

### Supplementary Information

**Additional file 1. **Muscle Quality Index is inversely associated with psychosocial variables among Chilean adolescents.

## Data Availability

The datasets generated and/or analysed during the current study are available in a supplementary file.
